# Degeneration of the osteocyte network in the C57BL/6 mouse model of aging

**DOI:** 10.18632/aging.101308

**Published:** 2017-10-26

**Authors:** LeAnn M. Tiede-Lewis, Yixia Xie, Molly A. Hulbert, Richard Campos, Mark R. Dallas, Vladimir Dusevich, Lynda F. Bonewald, Sarah L. Dallas

**Affiliations:** ^1^ Department of Oral and Craniofacial Sciences, School of Dentistry, University of Missouri Kansas City, Kansas City, MO 64108, USA; ^2^ Departments of Anatomy and Cell Biology and Orthopaedic Surgery, School of Medicine, Indiana University, Indianapolis, IN 46202, USA

**Keywords:** aging, osteocytes, osteoporosis, dendrite degeneration, bone fragility

## Abstract

Age-related bone loss and associated fracture risk are major problems in musculoskeletal health. Osteocytes have emerged as key regulators of bone mass and as a therapeutic target for preventing bone loss. As aging is associated with changes in the osteocyte lacunocanalicular system, we focused on the responsible cellular mechanisms in osteocytes. Bone phenotypic analysis was performed in young-(5mo) and aged-(22mo) C57BL/6 mice and changes in bone structure/geometry correlated with alterations in osteocyte parameters determined using novel multiplexed-3D-confocal imaging techniques. Age-related bone changes analogous to those in humans were observed, including increased cortical diameter, decreased cortical thickness, reduced trabecular BV/TV and cortical porosities. This was associated with a dramatic reduction in osteocyte dendrite number and cell density, particularly in females, where osteocyte dendricity decreased linearly from 5, 12, 18 to 22mo and correlated significantly with cortical bone parameters. Reduced dendricity preceded decreased osteocyte number, suggesting dendrite loss may trigger loss of viability. Age-related degeneration of osteocyte networks may impair bone anabolic responses to loading and gender differences in osteocyte cell body and lacunar fluid volumes we observed in aged mice may lead to gender-related differences in mechanosensitivity. Therapies to preserve osteocyte dendricity and viability may be beneficial for bone health in aging.

## INTRODUCTION

Aging is associated with osteoporosis, a disease of reduced bone mass and quality, which leads to increased fracture risk (reviewed in [1]). In the U.S., osteoporosis results in 1.5 million fractures per year [2] with costs projected to reach $20.3 billion annually by 2025 [3]. Approximately 40 million women in the U.S. have low bone mineral density (BMD) and increased fracture risk [2]. These fractures can lower quality of life and lead to chronic pain. Hip fractures are also associated with loss of mobility, loss of independence and the need for institutionalized care. Thus, under-standing the mechanisms behind bone loss and the changes in bone quality that occur with aging are critical in order to develop effective measures for prevention of osteoporosis and reduction of fracture risk in the elderly.

In both males and females BMD, trabecular bone volume and cortical bone thickness decrease with increasing age while cortical bone porosity increases, especially for post-menopausal women [2, 4, 5]. This bone loss results from imbalanced bone remodeling whereby bone formation is unable to replace the resorbed bone. In animal models, it has also been shown that trabecular bone volume, cortical bone thickness and bone strength decrease with age [6, 7] in a gender dependent manner [8] with females affected earlier and more severely than males.

The osteocyte plays a key role in maintenance of bone mass (for review see [9-11]). Sclerostin, which is expressed in osteocytes, inhibits Wnt/β-catenin signaling, a major pathway that regulates bone mass [12, 13]. Drugs targeting sclerostin inhibition have shown great promise in treating osteoporosis and reducing bone loss in clinical studies [14-17]. Additionally osteocytes are thought to regulate bone mass through their role in mechanotransduction and in coordinating adaptive responses to mechanical loading [18-25]. Osteocytes also regulate osteoclastic bone resorption through expression of M-CSF and RANKL [26-28]. Therefore, studying the osteocyte in its native environment and the degenerative changes that occur in osteocytes with aging are essential to increasing our understanding of their role in age related bone loss.

The majority of previous studies have used techniques that image the lacunocanalicular system (LCS) rather than the osteocyte network itself to infer age related changes in osteocytes and their dendrite connectivity. Confocal microscopy of basic fuschin staining has shown changes in the LCS with aging, such as decreased lacunar density [29]. Acid-etching/SEM of resin embedded bone (which provides a relief cast of the LCS) has shown decreased canalicular number with age in humans and rats [30, 31]. Similar results were found using Bodian stained sections in aged mouse femurs [32]. Studies of human bone indicate that a dense osteocyte network is associated with higher bone material quality [33] and decreased lacunar density correlates with microcrack accumulation [34], suggest-ing that the osteocyte network plays an important role in maintenance of healthy bone.

Recently, we have developed multiplexed confocal imaging methods combined with using fixable tracer dyes that allow us to simultaneously image several aspects of the osteocyte, including its cytoskeleton, nucleus, cell membrane and the LCS fluid space in 3D [35]. This has allowed us to examine alterations in the osteocyte network and dendrite connectivity directly without relying on extrapolation from imaging the LCS. Using these techniques, the aim of this study was to analyze directly for the first time the degenerative changes in osteocytes, their dendrite connectivity and their lacunar fluid space in young and aged mice and integrate these observations with age related histolo-gical and structural changes in the bones. Our data show dramatic differences in osteocyte connectivity, bone geometry and structure with aging that are gender dependent and may have important implications for mechanoresponsiveness and age related bone loss.

## RESULTS

### MicroCT shows changes in bone structure and geometry with aging

**Figure 1:**
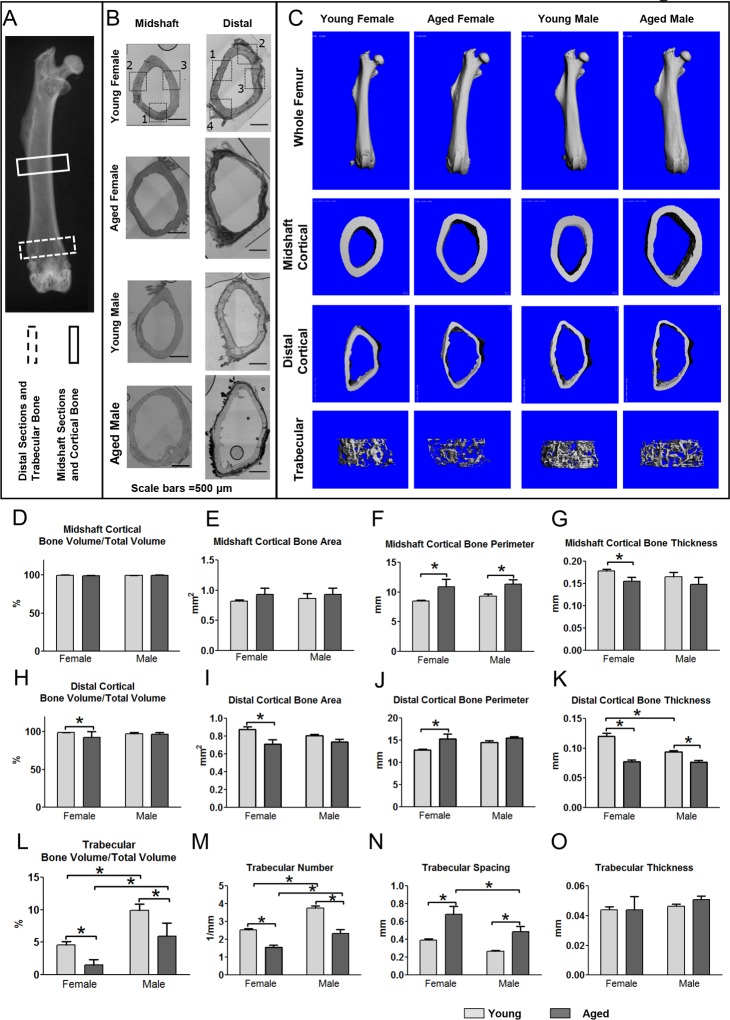
Aging is associated with structural changes in bone that are more pronounced in females than males (**A**) X-ray of mouse femur showing regions used for midshaft cortical and distal cortical and trabecular analysis. (**B**) Bright field montaged images of midshaft and distal sections used for confocal imaging of 5 and 22mo mouse femurs with imaging regions indicated by numbered black boxes (Bars = 500μm). (**C**) MicroCT reconstructions for whole femurs and midshaft cortical and distal cortical and trabecular bone. Graphs in (**D**-**G**) show microCT quantitation of midshaft cortical bone parameters, (**H**-**K**) show distal cortical bone parameters and (**L**-**O**) show quantitation of trabecular bone parameters in young and aged mice. (Data are mean ± SEM, * = p≤ 0.05, ANOVA/Tukey's, females n= 8, males n= 6-7) (Note: n=6 was used for the aged female group in graphs (**M**-**O**) because in two aged females the trabecular number was so low that the software cannot reliably compute values for trabecular spacing).

MicroCT analysis showed changes in femoral bone structure and geometry with aging. Reconstructed images of the whole bones and of cortical bone at the midshaft showed increased cortical diameter in aged compared to young femurs in both genders (Fig. 1C). Quantitative analysis at the midshaft showed no significant change in cortical bone volume/total volume (BV/TV), which is essentially a measure of cortical porosity, or cortical bone area (Fig. 1D, 1E). However, aging was associated with a significant increase in cortical bone perimeter of 28% in females and 23% in males (Fig. 1F) and a significant decrease in cortical bone thickness of 14% in females but not males (Fig. 1G). In aged males, there was considerable variation in cortical thickness around the bone perimeter, (Fig. 1C, second row, right panel), resulting in the difference in the average thickness being non-significant. Midshaft cortical bone parameters were not significantly different between genders.

**Figure 2:**
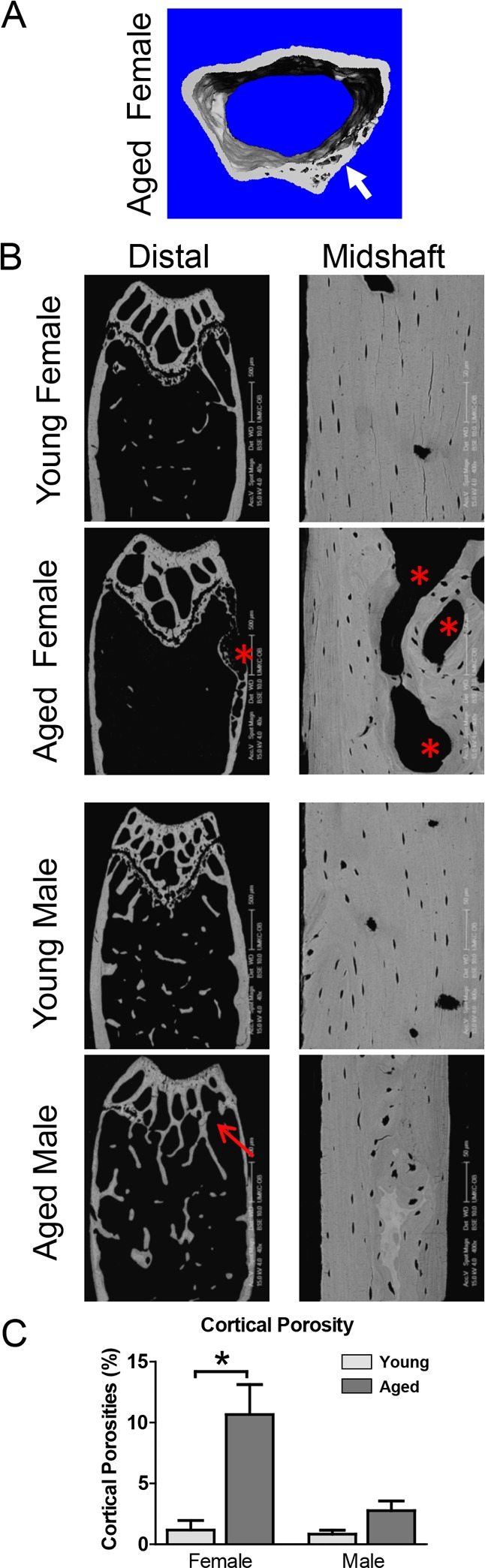
Aging is associated with cortical porosities, regional variation in mineral density and growth plate closure (**A**) microCT reconstruction of distal femur cortical bone in an aged female showing cortical porosity (arrow). (**B**) BSEM image of the distal femur (Bar = 500μm) and midshaft cortical bone (Bar = 50μm) in young and aged C57BL/6 mice. Arrow indicates growth plate closure in the aged male and * indicates cortical porosities. (**C**) Quantitation of cortical porosity from n=5 animals showing a significant increase in cortical porosity in female C57BL/6 mice with age.

Cortical porosities were observed in the femurs in several of the aged mice, particularly in females at the distal end (see Fig. 2A arrow and 2B asterisks). Therefore, an additional cortical analysis was performed on the distal femur. This showed that the distal cortical BV/TV was significantly decreased by 6.3% in females but not males (Fig. 1H), consistent with increased cortical porosities. Females but not males showed a significant 19% decrease in distal cortical bone area (Fig. 1I) and 20% increase in distal cortical bone perimeter (Fig. 1J). The reduced cortical thickness was more pronounced in the distal region than the midshaft, with a significant 36% reduction in females and a 19% reduction in males (Fig. 1K). Distal cortical bone parameters were not statistically significant between genders except for distal cortical bone thickness, which was significantly lower in males vs. females at 5mo (Fig. 1K).

Micro CT analysis of trabecular bone parameters showed that by 5mo, there was a significant gender difference in trabecular BV/TV, with males having ∼2-fold higher BV/TV compared to females (Fig. 1C, L). A significant reduction in BV/TV was seen with aging in both genders (66% in females and 40% in males) (Fig. 1C, 1L). This was due to decreased trabecular number and increased spacing (Fig. 1M, N) rather than changes in thickness (Fig. 1O). No significant differences were seen in BMD between genders or age groups in cortical or trabecular bone (data not shown).

### Backscattered SEM (BSEM) shows cortical porosities in aged mice and closure of the growth plate in aged males

BSEM confirmed reduced cortical thickness and expansion of cortical diameter in aged compared to young mice and showed cortical porosities in several of the aged mice, particularly females (Fig. 2B, asterisks).

This was more prevalent at the distal end of the femur. In all male mice, the growth plate was either completely or partially closed (Fig. 2B, arrow). BSEM also showed greater regional variability in bone density in aged mice, indicated by variation in greyscale intensity of the cortical bone (Fig. 2B). Quantitation of the cortical porosities revealed a significant increase in porosity in females but not males with age (Fig. 2C).

### Degenerative changes in osteocyte connectivity and density in aged mice

**Figure 3:**
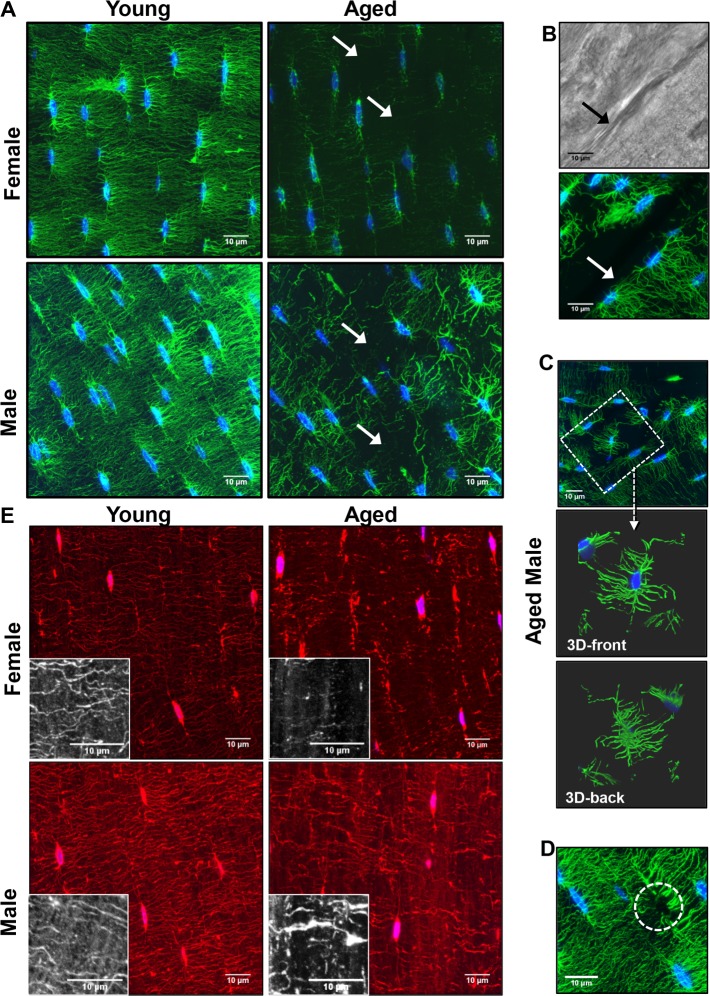
Degeneration of the osteocyte network in aged bone (**A**) Maximal Z-projections of 250 planes (32.5μm) from 100x confocal images of phalloidin (green) and DAPI (blue) stained midshaft femur sections showing osteocyte connectivity in young and aged male and female mice. (**B**) Dendrites do not cross over discontinuities in the bone (arrow) although matrix is present as seen in the corresponding brightfield image. (**C**) Some osteocytes in aged animals are found in “islands” with few or no connections to the surrounding osteocytes (dashed box). Enlarged images in (**C**) show a 3D render (front and back) of the same osteocyte confirming its lack of connectivity with surrounding osteocytes. (**D**) Occasionally dendrites with no visible cell body were seen in the aged mice (dashed circle) suggesting the dendrites may be left behind after apoptosis. (**E**) Maximal Z-projections of 20 planes (2.6μm) from DiI (red) and DAPI (blue) labeled femur sections showing staining of the osteocyte cell membrane in young and aged mice. Black and white insets show lipid material in the matrix around the dendrites. (Bars = 10μm).

Confocal imaging of phalloidin/DAPI stained thick sections was performed in the same cortical regions to determine how aging affects the osteocyte networks (Fig. 3A-D). This revealed a dramatic reduction in dendrite connectivity with aging in both genders (Fig. 3A and Supplementary Movies 1 and 2), which was more severe in females. Gaps were seen in the osteocyte network where an osteocyte would be expected to be located but was absent (Fig. 3A, arrowheads). We also observed “islands” of osteocytes disconnected from other osteocytes (Fig. 3B, C). These osteocyte “islands” were often located near discontinuities or cement lines as shown in Fig. 3B (arrow), suggesting remodeling. These data show not only reduced dendrite number but also reduced osteocyte connectivity with aging. Occasionally in aged animals, dendrites were seen without an osteocyte cell body (Fig. 3D, circle), suggesting the osteocyte had died, leaving its dendrites behind. Supplementary Fig. S1 shows additional confocal images of osteocytes in all three imaged regions of the cortex (see Fig. 1B for locations of imaged regions) to illustrate the regional variation in osteocyte dendrite connectivity.

Observations of phalloidin/DAPI staining were confirmed by confocal imaging using osteocyte cell membrane staining with DiI (Fig. 3E), showing that the observed reduction in dendrite number was not just due to decreased levels of actin in aged osteocytes. Imaging with DiI also showed decreased dendrite connectivity in aged mice that was more severe in females than males. The membrane dye also revealed extensive amounts of lipid material in the matrix that decreased in aged compared to young bone (Fig. 3E, insets). This lipid material is consistent with extracellular vesicle-like structures shed by osteocytes that we have described previously [35].

**Figure 4:**
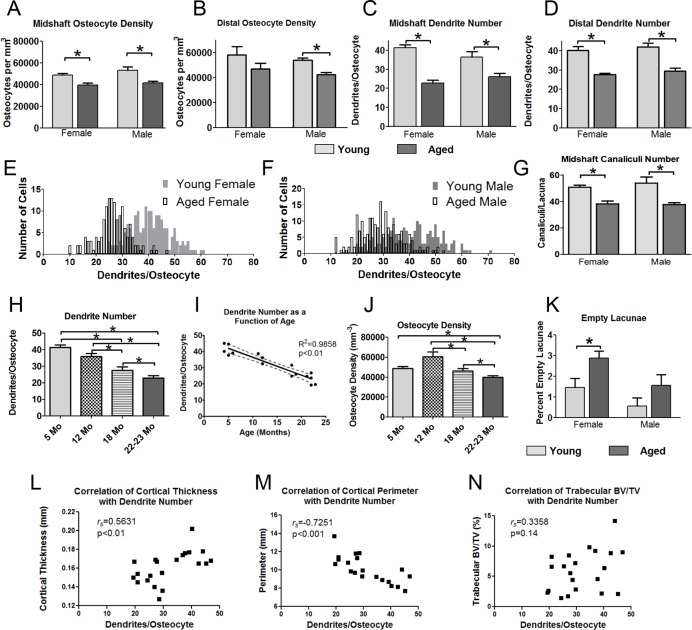
Aging is associated with reduced osteocyte dendrite connectivity, which precedes a decline in osteocyte number Quantitation of osteocyte density (**A**, **B**) and dendrite number per osteocyte (**C**, **D**) in the midshaft and distal femur from phalloidin stained sections in young and aged mice. (**E**, **F**) show frequency distribution plots of the number of dendrites per osteocyte in young and aged female (**E**) and male (**F**) mice. (**G**) Quantitation of the number of canaliculi per lacuna from Texas Red-dextran labeled midshaft femur sections in young and aged mice. (**H**) Time course showing a decline in dendrite number per osteocyte in female mice over 5, 12, 18 and 22mo. (**I**) Regression plot showing the linear decline in osteocyte dendrite number between 5 and 22mo in female mice. (**J**) Time course showing changes in osteocyte density in female mice at 5, 12, 18 and 22 mo. (**K**) Quantitation of empty lacunae in young and aged mouse femurs. (**L**-**N**) Scatterplots of osteocyte dendrite number versus cortical thickness (**L**), cortical perimeter (**M**) and trabecular BV/TV (**N**) with Spearman's correlation coefficient and significance indicated. (Data are mean ± SEM, * = p≤ 0.05, ANOVA/Tukey's) (**A**-**D** and **K**), females n=5, males n=6; G, n=5; (**H**, **J**, n= 3-5).

Quantitation of confocal phalloidin/DAPI stained image stacks showed a significant reduction in osteocyte number per mm^3^ of 18% in females and in 27% in males in the midshaft (Fig. 4A). A significant 21% decrease was seen for males in the distal region as well, with females trending downwards (Fig. 4B). Differences in osteocyte density between genders were not significant. Accompanying decreased osteocyte density, there was a dramatic decrease in dendrite number per osteocyte in aged mice (Fig. 4C, D). The effect was more severe in females (45% reduction) compared to males (27% reduction), particularly in the midshaft, however, a significant decrease was also found for both genders in the distal region. Frequency distribution plots of dendrite number per osteocyte (Fig. 4E, F) showed that in females the osteocyte populations in young and aged mice were more clearly separated than in males. Male mice had a wider distribution of dendrites per cell compared to females and in both genders there was a shift in the population towards lower numbers of dendrites per osteocyte with aging. Additionally, the number of canaliculi per osteocyte was quantified from confocal image stacks in mice injected with fixable 10kDa Texas Red-dextran and stained with phalloidin/DAPI. The Texas Red-dextran permeates the lacunar and canalicular fluid space and can be used to image the canaliculi and lacunae [35].

This revealed that the number of canaliculi per lacuna in the midshaft also decreased significantly with age, by 26% in females and 30% in males (Fig. 4G). Interestingly, the canaliculi outnumbered primary dendrites by 1.2-1.4 fold in the young mice and by 1.5-1.7 fold in the aged mice, showing that not every canaliculus is occupied by a dendrite.

To further understand the kinetics of the decline in osteocyte density and dendrites, an extended time course of 5, 12, 18 and 22mo was analyzed in females (Fig. 4H). This showed a linear decline in dendrite number per osteocyte from 5 through 22 mo. A linear regression plot of age against dendrite number con-firmed a highly significant negative linear correlation (R^2^= 0.9858, p<0.01) (Fig. 4I). Interestingly, osteocyte number per mm^3^ bone peaked at 12mo (Fig. 4J), when dendrite number had already started to decline (Fig. 4H). The osteocyte density then steadily declined from 12 to 22 mo. Therefore the decline in dendrite number preceded the decline in osteocyte number. The number of empty osteocyte lacunae was measured in un-decalcified plastic sections of the femur (Fig. 4K). The overall percentage of empty lacunae was low (≤3% in all groups) and increased significantly with age in females, doubling from ∼1.5% to 3%. Males did not show a significant increase in empty lacunae, which may be partly because the percentage started out low (less than 1%) in young males. Fig. 4L-N show scatter plots of osteocyte dendrite number versus cortical thickness, cortical perimeter and trabecular BV/TV. Spearman correlation tests showed a significant positive correlation between dendrite number and cortical thickness (*r_s_* = 0.5631, p<0.01) and a negative correla-tion between dendrite number and cortical perimeter (*r_s_* = −0.7251, p<0.001), with no significant correlation between trabecular BV/TV and dendrite number.

### Changes in osteocyte cell body and lacunar volume with age

**Figure 5:**
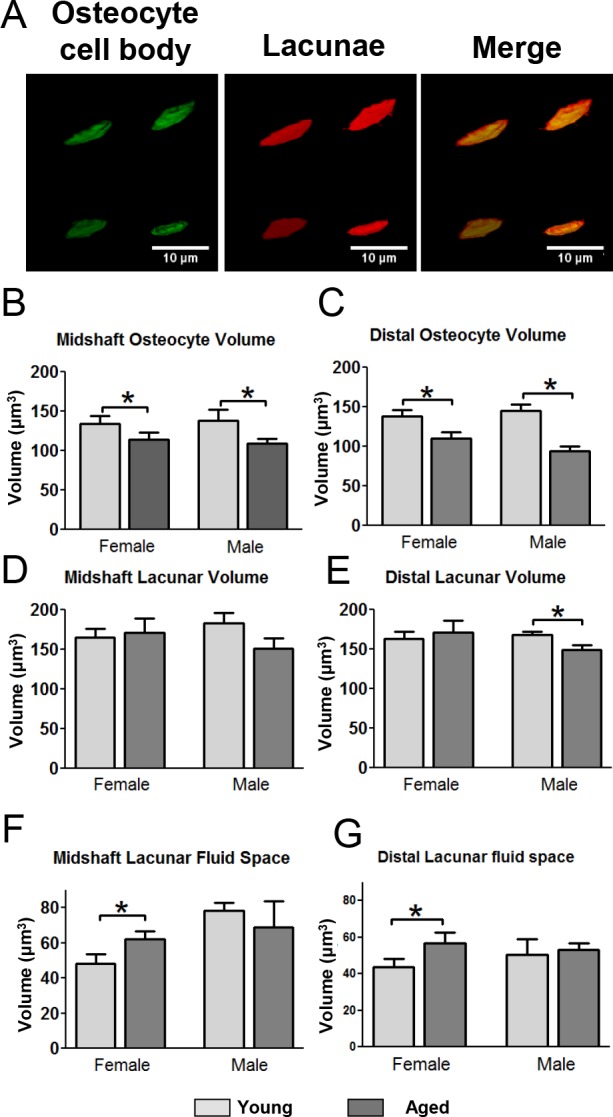
Decreased osteocyte cell volume with decreased lacunar volume in aged males but not females leads to gender differences in lacunar fluid volumes (**A**) Examples of 3D renderings of the osteocyte cell bodies labeled with DiO and lacunae labeled with fixable Texas-Red-dextran for volumetric calculations. (**B** and **C**) quantitation of osteocyte cell body volume in the midshaft (**B**) and distal (**C**) region. (**D** and **E**) quantitation of the corresponding lacunar volumes. (**F** and **G**) show calculation of the lacunar fluid space derived from subtracting the osteocyte cell body volume from its corresponding lacunar volume. (Data are mean ± SEM, * = p≤ 0.05, ANOVA/Tukey's; n= 5).

In mice injected with fixable Texas Red-dextran and stained with the membrane dye DiO, confocal image stacks were used to create 3D volumes of the osteocyte cell body and its corresponding lacuna (Fig. 5A). Quantitative analysis showed that the osteocyte cell body volume in female and male mice decreased significantly with age (15% and 19%, respectively) (Fig. 5B) in the midshaft with a similar reduction in the distal region (Fig. 5C). While the cell body volume decreased in aged female mice the average lacunar volume was not significantly changed (Fig. 5D, E). In contrast, males showed a small decrease in lacunar volume with aging which was significant in the distal region (Fig. 5D, E). This results in an increased lacunar fluid space in female mice but no change in males with aging (Fig. 5F, G).

### Histology reveals cortical porosities, loss of growth plate in males and altered osteoblast and osteoclast activity in aged animals

**Figure 6:**
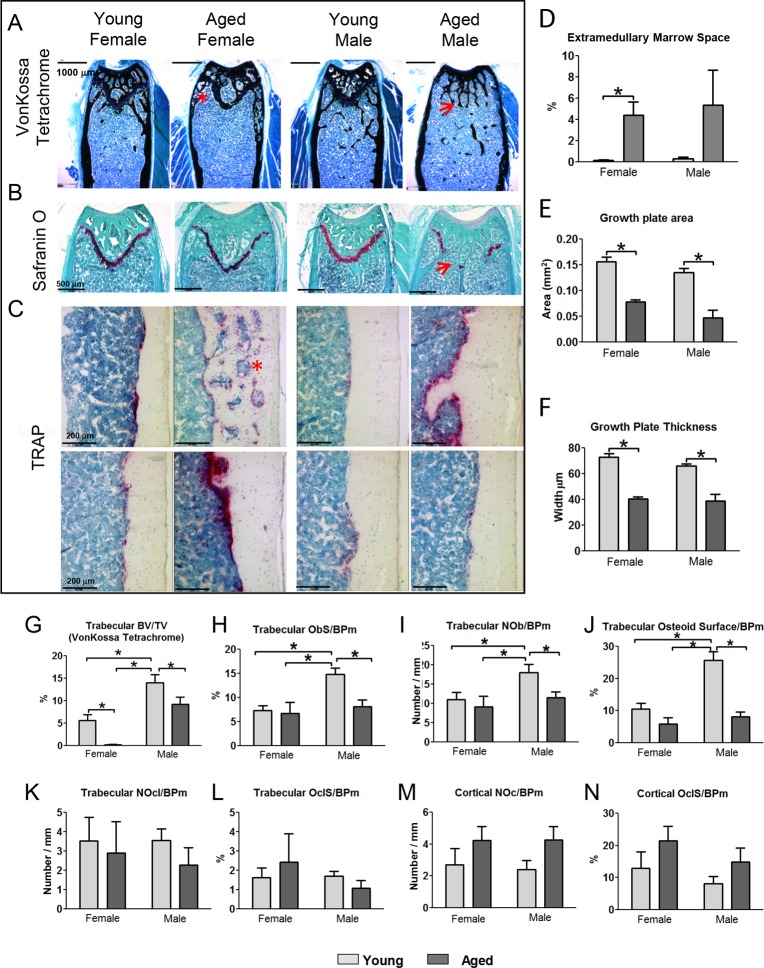
Histomorphometry shows increased extramedullary marrow, decreased osteoblast activity and decreased growth plate area and thickness in aged mice (**A**) von Kossa Tetrachrome stained sections (Bar = 1000μm) and (**B**) Safranin O stained sections (bar = 500μm) from the distal femur in young and aged mice. (**C**) TRAP stained sections at the midshaft. The images on two rows show different individual mice to illustrate the variability of TRAP staining in aged mice (Bar = 200μm). Arrows indicate regions where the growth plate has closed and * indicates extramedullary marrow spaces. (**D**) Quantitation of the extramedullary marrow space area, (**E**) growth plate area and (**F**) growth plate thickness from von Kossa Tetrachrome stained sections in young and aged mice. (**G**-**L**) show histomorphometric quantitation of osteoblast and osteoclast parameters in femoral trabecular bone of young and aged mice. (**M** and **N**) show histomorphometric quantitation of osteoclast parameters in cortical bone of young and aged mice. (Data are mean ± SEM, * = p≤ 0.05, ANOVA/Tukey's; females n=5, males n=6).

Histology using von Kossa tetrachrome staining confirmed the age related loss of femoral trabecular bone and expansion of cortical diameter in males and females (Fig. 6A). Loss of the growth plate was apparent in all 6 aged male femurs with both von Kossa Tetrachrome and Safranin O staining and ranged from partial to complete loss (Fig. 6A, B, arrow). In contrast, all females retained the growth plate although it was thinner with aging (Fig. 6B). Histological examination of the cortical porosities revealed the presence of extramedullary marrow (Fig. 6A, C, asterisks). TRAP staining suggested increased numbers of osteoclasts in male and female aged mice (Fig. 6C), which was particularly prominent in extramedullary marrow spaces associated with cortical porosities (asterisk). However, the TRAP staining in aged mice was variable between animals (Fig. 6C).

The area of extramedullary marrow spaces (marrow filled cortical porosities) was measured as a percentage of total bone area. A significant increase in extra-medullary marrow spaces was seen in females with age (Fig. 6D). Males trended towards an increase that did not reach statistical significance due to the variability in aged males. Measurements from safranin O stained sections showed a significant decrease in growth plate area and thickness in both genders (Fig. 6E, F). No significant differences between genders were seen although males showed a larger reduction in growth plate area than females, due to complete or partial loss of the growth plate in males.

Histomorphometric analysis in von Kossa tetrachrome stained sections showed a significant loss of trabecular BV/TV in young vs. old mice of both genders (Fig. 6G) in agreement with microCT data. The number of osteoblasts per bone perimeter (Ob/B.Pm), osteoblast surface per bone perimeter (ObS/B.Pm), and osteoid surface/B.Pm in trabecular bone were highest in young male mice and were significantly reduced in aged males (Fig. 6H-J). Females already had significantly lower values for all these trabecular osteoblast parameters than males at 5mo and showed no additional significant change with aging (Fig. 6H-J). No significant differences were observed in osteoclast number (NOcl/B.Pm) or osteoclast surface (OCLS/B.Pm) in the trabecular bone compartment (Fig. 6K-L). In the cor-tical bone there was a significant increase in the total number of osteoclasts in aged male mice (from 36.33±10.48 at 5mo to 105.9±36.81 at 22mo, p<0.05) but not females (58.20±30.97 at 5mo and 161.0±59.15 at 22mo). However, since much of this osteoclast activity was associated with remodeling within the cortical porosities, the bone surface was also increased and therefore differences in the NOcl/B.Pm and OCLS/B.Pm did not reach significance (Fig. 6M, N).

### Aging in the C57BL/6 mouse model is associated with calcification of the achilles tendons

Radiographs of the hindlimbs showed that calcification of both Achilles tendons occurred in 100% of male and female aged mice. In contrast, none of the 5mo old mice showed tendon calcification (Supplementary Fig. S2, arrows).

## DISCUSSION

In this study, a comprehensive analysis of age related changes in bone was performed using the C57BL/6 aged mouse model. Using newly developed multiplexed 3D confocal imaging methods and fixable tracer dyes we have for the first time simultaneously imaged and quantified osteocytes and their dendrite connectivity directly, together with their lacunocanalicular networks in aged mice and correlated these with changes in bone structure and geometry analyzed by microCT and histo-morphometry. Our data show that aging is associated with changes in bone structure and geometry in the femur including increased cortical diameter, decreased cortical thickness, reduced trabecular BV/TV and cortical porosities resulting in extramedullary marrow spaces. These changes are analogous to degenerative age related changes in humans [2, 4, 36, 37] in which cortical thinning, reduced trabecular bone volume and cortical porosities are seen. These age related changes were gender specific in C57BL/6 mice, with females showing a more dramatic loss in trabecular bone and more cortical porosities compared to males and males showing closure of the growth plate with aging. The increased femoral cortical diameter with aging would lead to a greater moment of inertia in aged bones and likely represents an adaptive response to maintain bone strength in a bone with thinner cortices.

Other studies have used microCT to evaluate age related changes in bone architecture in C57BL/6 mice in males [6] or both genders [8] but have not examined both midshaft and distal cortical bone or integrated this with simultaneous analysis of the osteocyte lacunocanalicu-lar network and histomorphometry. Our results are in agreement with these microCT studies, which report reduced trabecular BV/TV, increased cortical bone perimeter and decreased cortical thickness with more pronounced effects in females than males. In a study of PTH effects on skeletal aging in C57BL/6 mice and its effects to antagonize age-related oxidative stress, similar changes in bone architecture in the femur and vertebrae were reported in female aged mice [7].

A key goal of this study was to determine directly how aging alters the osteocyte network and its connectivity. Previous studies have imaged the LCS to infer age related changes in the osteocyte network [29, 30]. While this has yielded useful information, looking at the LCS may not distinguish between occupied vs. empty lacu-nae, and this indirect approach assumes that all canaliculi are occupied by a dendrite, which may lead to overestimation of osteocyte and dendrite numbers. Our previously developed methods for multiplexed 3D confocal imaging of osteocytes and their LCS [35] were employed to determine directly how the osteocyte network is altered in C57BL/6 aged mice. We show a 45% decrease in midshaft osteocyte dendrite number in aged female mice and a 27% decrease in males. Coupled with reduced dendricity was a decrease in osteocyte density of 18% in females and 27% in males. From these data it can be inferred that the combined effect would be an overall reduction in connectivity of the osteocyte network of ∼55% in females and ∼47% in males. The dendrite number we observed per osteocyte in young mice (∼40 dendrites/cell) is well within ranges reported by others in various species [30, 38, 39]. Moreover, many of these studies examined canaliculi rather than dendrites and may overestimate the actual number of dendrites. Indeed, our quantitations underscore that the number of canaliculi is about 1.2 – 1.4 fold higher than the number of primary dendrites in young mice and 1.5 - 1.7 fold higher in aged mice, showing that not all canaliculi are occupied by a dendrite and that the occupancy rate may decline with aging.

Decreased numbers of canaliculi/lacunae have been shown with aging in mice between 3mo and 2 years of age [32] and in humans [30], consistent with our findings. Importantly, our study revealed a linear decline in dendrites between 5, 12, 18 and 22mo in female C57BL/6 mice, with a negative linear correlation between age and dendrite number. Dendrite number also correlated with cortical thickness and was negatively correlated with cortical bone perimeter, suggesting that maintaining osteocyte connectivity may be important for preventing resorption of bone on the endosteal surface and for limiting periosteal expansion. This could potentially be through regulation of osteocyte RANKL at the endosteal surface and sclerostin at the periosteal surface, but further studies are needed to determine the mechanism. Interestingly, the decrease in osteocyte dendricity preceded the decline in osteocyte number, suggesting that dendrite loss could be a trigger for loss of osteocyte viability. Therefore therapeutic interventions aimed at preserving or regenerating osteocyte dendrites may be beneficial for osteocyte viability and bone health. It remains unclear if osteocyte dendrite loss is irreversible, but our data raise the possibility that new dendrites could be extended into canaliculi that are unoccupied. In addition, some studies suggest the potential for regeneration, since osteocytes can remove mineral and remodel their local environment [40, 41] and can continue to extend dendrites into the matrix after dif-ferentiation [42]. This is achieved through the expression of genes related to osteoclastic resorption (but not differentiation), such as TRAP, cathepsin K and vacuolar proton pump subunits, ATP6V0D2 and ATP6V1G1, as well as matrix metalloproteinases 13 and 14 [40-42].

Osteocytes are not the only cells showing dendrite loss with age. In neuronal cells dendrite pruning is associated with aging and impaired nervous system function in a variety of disorders including Parkinson's disease, amyotrophic lateral sclerosis, Alzheimer's disease and ischemic brain injury [43, 44]. Many neuronal disorders with dendrite loss have been linked to autophagy dysfunction [45, 46] and a recent study reported that mice with osteocyte targeted knockout of Atg7 (a protein essential for autophagy) show an aging bone phenotype with reduced trabecular BV/TV, reduced cortical thickness and increased cortical porosity by 6 months [47]. An intriguing possibility is that neurodegenerative and osteocyte degenerative conditions may share common mechanisms. One potential therapy showing promise in animal models is the anti-epileptic and anti-Parkinson's disease drug Zonisamide, which enhances neurite elongation in primary motor neurons and increases axon size in sciatic nerve autograft in mice [48]. Similar approaches could be studied as a means to preserve osteocyte connectivity and potentially improve bone homeostasis.

The age related reduction in osteocyte connectivity has several implications. As osteocyte dendrites rather than the cell body are thought to be critical for mechano-transduction [49] reduced dendrite connectivity may contribute to the impaired bone response to mechanical loading in aged animals [50]. Reduced osteocyte density has been associated with accumulation of microdamage and microcracks [34], which could be another mechanism contributing to skeletal fragility. In addition to reduced osteocyte and dendrite number, aged bones had more interfaces (cement lines from bone remodeling) and increased cortical porosity, most likely due to intracortical remodeling. This further reduced osteocyte connectivity by creating “islands” of osteo-cytes that were not connected to other osteocytes. Decreased osteocyte connectivity could lead to a downward spiral in viability as essential nutrients are obtained though the canalicular network and important signals are transferred through cell-cell contact. As more osteocytes are lost and connectivity is reduced, this may lead to further stress on the remaining osteocytes and further loss of viability.

In this study the osteocyte density decreased with aging but this decrease was not fully accounted for by increased empty lacunae. The number of empty lacunae in young mouse femurs was very low (1.5% or less) and although it increased 2-fold in aged females, was never higher than 3%. Other groups have measured empty lacunae in paraffin sections and reported numbers from around 22-23% in young and 32-34% in aged mice. We performed similar analyses in paraffin sections and also found higher percentages of empty lacunae (data not shown). In our experience, quantifying from paraffin sections greatly overestimates the numbers of empty lacunae, due to shrinkage of osteocytes during processing, and the values from plastic sections are more accurate. Since the age-related reduction in osteocyte number is not accounted for by empty lacunae, one possibility is that micropetrosis fills in lacunae after osteocyte apoptosis, which is supported by studies in human bone [36].

Most previous studies of osteocyte connectivity have used acid etch SEM imaging, nanoCT or synchrotron X-ray methods that image the lacunae and canaliculi but not the osteocytes themselves. Using multiplexed confocal imaging together with fixable tracer dyes we can simultaneously measure the osteocyte cell body and lacunar volume in young and aged mice, which was previously only possible by TEM. Our data suggest that female osteocytes “shrink” within their lacunae by reducing their cell body but not lacunar volume, resulting in an increased lacunocanalicular fluid volume. In contrast, male osteocytes reduced both their cell body and lacunar size with no significant change in lacunar fluid volume. These gender related differences in fluid volumes could lead to differences in mechano-sensitivity, as the patterns of lacunar and canalicular fluid flow shear stress may be different in males and females. Our multiplexed confocal imaging provides a more comprehensive picture of age-related degenerative changes in osteocytes and their LCS networks and such data can now be used to create more sophisticated computational models of bone loading and the local strain environments around osteocytes as well as how this is altered with aging.

In the current study, we saw the appearance of large cortical porosities with aging, particularly in female mice. These were not reported in other microCT studies of skeletal aging in C57BL/6 [6, 8]. However, cortical porosities were observed in aged female C57BL/6 mice by Jilka et al. [51]. They further showed that dys-apoptosis of osteocytes by targeted deletion of BAK and BAX, two genes essential for apoptosis, increased cortical porosity suggesting that signals from old or damaged osteocytes may be responsible. These cortical porosities were preferentially localized to the endosteal region in Jilka's study and ours and are analogous to increased cortical porosity in humans with aging [4, 52]. However, in humans the porosities occur due to increased size of Haversian canals whereas mice are not thought to form Haversian bone. Histologically, the cortical porosities in our mouse model contained marrow. Mouse cortical bone has a network of blood vessels running through it [53] and it is likely that these extramedullary marrow spaces initiate around blood vessels, resembling Haversian remodeling in humans.

By histomorphometry, we saw a significant decrease in trabecular osteoblast number and osteoblast surface per bone surface in aged males. This was not true for females, most likely because there was so little trabecu-lar bone in the femur in aged females that it is difficult to obtain robust estimates of osteoblast number and surface. Interestingly, Almeida et al. examined the vertebrae in aged C57BL/6 mice, which retain more of their trabecular bone compared to the femur, and showed a significant reduction in osteoblast number in 16, 25 and 31 month old female mice compared to 8 month controls [54].

Another novel observation from our study was that in aged C57BL/6 males but not females, the growth plates were completely or partially closed, implying that by 22mo male mice might have a limited capacity to generate new trabecular bone when subjected to stimuli such as exercise or bone anabolic agents. Interestingly, 100% of the male and female mice showed calcification of the Achilles tendon. This would likely impair muscle function/ambulation, which could in turn affect bone loading, muscle contractile strength and/or muscle-bone crosstalk.

In summary, the current study shows gender specific changes in the aging mouse skeleton, including a decline in femoral trabecular bone volume, cortical thickness and increased cortical diameter. These changes were accompanied by dramatic reductions in osteocyte dendrite connectivity that were more pronounced in females and declined linearly with age. Loss of dendrites preceded a reduction in osteocyte cell density, suggesting that dendrite loss may be a trigger for loss of osteocyte viability. Males and females also showed differences in the size of the osteocyte cell body relative to its lacuna with aging, leading to potential differences in strain induced fluid flow shear stress patterns between males and females. Future studies are needed to focus on approaches for preserving osteocyte connectivity with aging, such as exercise intervention, agents that target the autophagy pathway and potentially approaches previously used to regenerate neuronal dendrites. This could lead to new therapies to prevent degeneration of the osteocyte network and preserve bone homeostasis in the aged skeleton.

## METHODS

### Animals

The C57BL/6 mouse model of aging was used in which 5 month old (5mo) mice represent young adults and 22-23mo old mice represent aged (geriatric) adults [6, 51]. 12 and 18mo mice were used in some experiments to model early aging. Mice of both genders were obtained from the NIH/NIA aged rodent colony (sample sizes for experiments are indicated on the accompanying figure legends). Animal experiments and euthanasia were performed under an approved IACUC protocol at the University of Missouri Kansas City (UMKC), and conformed to relevant federal guidelines. The UMKC animal facility is AAALAC approved and animal care and husbandry meets requirements in the Guide for the Care and use of Laboratory Animals (8th Ed.), National Research Council. Animals were group housed and maintained on a 12hr light/dark cycle with *ad libitum* food and water at 22°C constant temperature and 45-55% humidity.

### Bone sample preparation

Unless stated otherwise, reagents and chemicals were purchased from Sigma Aldrich, St. Louis, MO. Mice were humanely euthanized and the femurs were fixed in 4% paraformaldehyde (PFA) in PBS, pH 7.4 at 4°C for 24hr. One femur was stored in 70% ethanol at 4°C prior to microCT analysis (see below). After microCT, the undecalcified femurs were dehydrated in graded ethanols. They were then infiltrated with acetone, then 1:1 followed by 1:2 acetone with methyl methacrylate infiltration solution [84% methyl methacrylate (MMA), 14% dibutyl phthalate, 1% polyethylene glycol, 0.7% benzoyl peroxide], and then 100% MMA for 5 days before plastic embedding in MMA with 0.033% N,N-dimethyl p-toluidine at −20°C for 3-5 days. The other femur was prepared for cryosectioning as described previously [35]. Briefly, after PFA fixation the femurs were decalcified in 10% EDTA, pH 7.4 for 1-2 wks, then equilibrated in 15% then 30% sucrose in PBS before embedding and freezing in Tissue-Tek O.C.T compound (Sakura Finetek USA Inc., Torrence CA).

### MicroCT

MicroCT analysis was performed with a vivaCT40 (Scanco Inc, Basel, Switzerland) in accordance with recommended guidelines [55] using an X-ray energy of 55kV (145μA), a voxel resolution of 10.5μm, 200ms integration time with the number of projections set at 1000/180 degrees and using a 0.5mm aluminum low pass filter. The threshold was set to 316 (equivalent to 498.5 mgHA/ccm) for both cortical and trabecular bone to distinguish mineralized from non-mineralized tissue. Two VOIs were chosen for cortical bone analysis. First, 50 slices (525μm) were contoured in the midshaft starting at the distal end of the third trochanter and progressing toward the knee joint. Second, 100 slices (1050μm) were contoured starting at the metaphyseal end of the distal growth plate, below the primary spongiosa, and progressing toward the midshaft. This same VOI was used for contouring of trabecular bone with cortical bone excluded. Bone properties were analyzed using Scanco bone evaluation software.

### 3D Multiplexed confocal imaging

For 3D multiplexed confocal imaging of osteocyte networks, 50μm transverse sections were cut from de-calcified O.C.T. embedded femurs on a Leica CM3050S cryomicrotome (Leica Microsystems, Wetzlar, Germany). Sections were taken from the midshaft and distal regions as shown in Fig. 1A and stained en block. For full details of multiplexed staining methods see [35]. Briefly, to stain the actin cytoskeleton, sections were incubated in 165nM AlexaFluor-488-phalloidin (ThermoFisher Scientific, Waltham, MA) in PBS overnight followed by three PBS washes then a 30min incubation in 4μg/ml DAPI (ThermoFisher) in PBS. Stained sections were coverslip mounted in 1:1 glycerol:PBS with 1mM MgCl_2_. For visualization of osteocyte membranes, sections were incubated for 2 days in 1,1′-dioctadecyl-3,3,3′3′-tetramethylindocarbo-cyanine perchlorate (DiI) or 3,3′-dioctadecyloxacarbo-cyanine perchlorate (DiO) at 100μM (ThermoFisher) in 9:1 ethanol:DMSO. Sections were washed briefly in ethanol followed by three PBS washes prior to labeling with DAPI as above. DiI or DiO labeled sections were mounted in 97% TDE (2,2′-thiodiethanol) mounting buffer as described previously [35], which matches the refractive index of the tissue samples to the immersion oil, thereby minimizing light spreading in the Z-axis. For experiments in which the LCS was imaged simultaneously with the osteocyte cell membrane, mice were injected intravenously with a Texas Red conjugat-ed lysine fixable 10kDa dextran (ThermoFisher) (32 mg/kg in PBS) 4 min prior to euthanization. This dye distributes into the lacunae and canaliculi and is fixable with aldehyde based fixatives, so it survives decalcification.

For confocal imaging, two sections per animal for each type of staining were imaged on a Leica TCS Sp5 II confocal microscope (Leica Microsystems, Wetzlar, Germany) in resonant scanner mode with line averaging set to 96. A tiled image of the entire transverse section was obtained with the 5x objective (NA 0.15 Zoom 1.7) followed by collecting detailed Z-stacks of 250-350 Z-planes with the 100x oil objective (NA 1.44 zoom 1.7 with a 0.13 μm step size) from three standardized regions in the midshaft sections and 4 regions in the distal sections as indicated in Fig. 1B. Using tranverse sections means that the osteocytes are imaged with their longest axis as the Z-axis. For phalloidin imaging 488nm laser excitation was used with an emission collection window of 493-580 nm acquired together with a brightfield image. For imaging DiI, laser excitation was 543 nm with a collection window of 553-650 nm. For DiO/Texas Red-dextran dual imaging the DiO was excited at 488 nm with an emission window of 494-564 nm and Texas Red was excited in the same scan at 594 nm with a 604-684nm collection window. In all confocal experiments DAPI signal was acquired in a separate scan using 405nm laser excitation and a collection window of 410-480 nm.

### Quantitation of 3D osteocyte parameters

For determination of osteocyte density, 3D reconstructions were generated of phalloidin/DAPI stained 100x image stacks with 250 slices (32.5 μm tissue depth) (see Supplementary Movies 1 and 2). These 3D reconstructions were used to count osteocyte number in the 272,217 μm^3^ volume using ImageJ software (Rasband, W.S., ImageJ, U. S. National Institutes of Health, Bethesda, MD, http://imagej.nih.gov/ij/, 1997-2015) with the cell counting plugin (Author: Kurt De Vos, University of Sheffield Academic Neurology). To avoid over or under estimation of osteocyte numbers, osteocytes caught in partial section at the edges of the 3D volume were included in the count on only three of the six faces of the cube volume. The mean number of osteocytes per mm^3^ of bone was determined for each animal in both midshaft and distal regions.

To measure the number of dendrites per osteocyte, 100x stacks of phalloidin labeled sections were contrasted in ImageJ to visualize the dendrites in the entire stack. Dendrites were only counted if the entire osteocyte cell body was captured in the Z stack and all dendrite sprouting points at the cell body could be discerned. Using the Image J Cell Counting plugin, dendrites were counted by scrolling through the Z stack and counting each dendrite at its sprouting point from the cell body. At least 60 osteocytes were counted per animal (i.e. per data point) for both midshaft and distal regions to obtain the average number of dendrites per osteocyte for each animal. Quantitation of canaliculi per lacuna was performed in a similar manner using stacks with Texas Red dextran labeling of the lacunocanalicular system.

Image stacks from dextran, DiO and DAPI multiplexed imaging were used for simultaneous determination of lacunar and cell body volumes. To determine osteocyte cell body volume RGB image stacks of DiO images merged with DAPI were converted to 8 bit greyscale images to create a “filled in” image of the intact osteocyte. The 8 bit stacks were then normalized to correct for signal loss with tissue depth, and thresholded in the Object Counter 3D plugin [56] so that just the cell body was measured and not cell processes. A lower volume limit of 10 μm^3^ was used. Numbered volumes were inspected in a 3D projection and volumes not representing an intact osteocyte (i.e. partially sectioned osteocytes) were excluded. Lacunar volumes were determined using the same method from dextran/DiO/

DAPI greyscale images. By subtracting the lacunar volume from its corresponding cell body volume a fluid space volume was determined for each lacuna. Only lacunae occupied by an osteocyte were measured and 40-60 lacunae were measured for both the midshaft and distal regions to determine the average cell body and lacunar size for each animal.

### Histology

Undecalcified longitudinal plastic sections of the femur (5μm thickness) were cut on a Microm HM 355S microtome with tungsten carbide blade. After the sections were deplasticized and rehydrated, von Kossa tetrachrome staining was performed using standard histological procedures. Histochemical staining for TRAP was done using standard procedures with an extended incubation time of 5hrs at 37°C in Naphthol AS-BI Phosphate substrate and using toluidine blue as the counterstain. Safranin O staining was performed with Fast Green counterstaining using standard histological staining methods. Stained sections were coverslip mounted in permount.

### Histomorphometry

For all histomorphometric quantitations, three non-consecutive longitudinal sections were measured per animal and mean values from these sections used as the data point for one animal. Osteoblast and osteoclast quantitation was performed on von Kossa Tetrachrome or TRAP stained sections, respectively using a Nikon E800 microscope (Nikon Instruments, Inc. Melville NY) and SONY Exwave HAD camera (Sony Corp., New York, NY) interfaced with an OsteomeasureXP bone histomorphometry system (OsteoMetrics Decatur, GA). Measurements were made from trabecular bone in the secondary spongiosa at a standard location 80μm below the growth plate extending ∼450μm using a 20x objective. The following parameters were measured directly or derived from measured indices: bone volume per total volume (BV/TV), osteoblast number, osteo-blast surface and osteoid surface per bone surface (N.Ob/B.Pm, Ob.S/B.Pm, OS/B.Pm), osteoclast number and osteoclast surface per bone surface (N.Ocl/B.Pm, OclS/B.Pm). For quantitation of empty osteocyte lacunae and TRAP positive osteoclasts in cortical bone, three non-consecutive longitudinal TRAP-stained femur sections were used per animal. For each section, twelve images were taken of the central midshaft, six from the medial and six from the lateral cortex using a 20x objective. The number of empty lacunae and lacunae occupied by an osteocyte were counted using the Cell Counter Plugin in ImageJ. Lacunae with a long axis <5μm were excluded as they represent sections that caught the outer edge of a lacuna and it could not be reliably determined if they were occupied. Osteoclast parameters for cortical bone were measured using the Cell Counting plugin and Measure functions in ImageJ.

### Backscattered SEM (BSEM)

For BSEM, the MMA embedded blocks remaining after sectioning for histology were polished with graded sandpapers (600, 800, 1200 grit [Buehler, Lake Bluff IL]) followed by Metadi Supreme Polycrystalline Diamond Suspensions of 1, 0.25, and 0.05μm (Buehler). Observation of backscattered electrons was performed on a field-emission SEM XL-30 scanning electron microscope (FEI, Hillsboro, OR) equipped with a solid state BSE detector on Au-Pd coated specimens using 15.0kV energy at 40x and 100x magnification. Quantitation of the cortical porosity as a percentage of the bone volume was done from 40x BSEM images of the distal region of the femur in ImageJ and using the measure analysis tool to determine the areas.

### Statistical analysis

For statistical comparisons, one way analysis of variance (ANOVA) was used followed by Tukey's post hoc test. Linear regression and Spearman's Correlation tests were used to test for significant correlations between datasets. Data were considered statistically significant with a p value ≤0.05.

## SUPPLEMENTARY MATERIAL FIGURES AND MOVIES






