# Supplementary material for: Degeneration of the osteocyte network in the C57BL/6 mouse model of aging
Source: Aging (Albany NY). 2017 Oct 26;9(10):2190–208. doi: 10.18632/aging.101308 (PMC5680562; doi:10.18632/aging.101308)
Supplement: Supplementary file 1 [file aging-09-2190-s001.pdf]

SUPPLEMENTARY MATERIAL

Please browse the Full text version of this manuscript to see the Supplementary Movies:

Supplementary Movie 1. Movie showing osteocyte networks in 3D rotation from the femoral midshaft of a young and aged female mouse (3D render of DAPI/phalloidin stained osteocytes, 250 planes, imaged at 100x magnification). Note the dramatic reduction in 3D osteocyte connectivity in aged female mice. Bar = 10µm .

Supplementary Movie 2. Movie showing osteocyte networks in 3D rotation from the femoral midshaft of a young and aged male mouse (3D render of DAPI/phalloidin stained osteocytes, 250 planes, imaged at 100x magnification). Note the dramatic reduction in 3D osteocyte connectivity in aged male mice. Bar = 10µm.

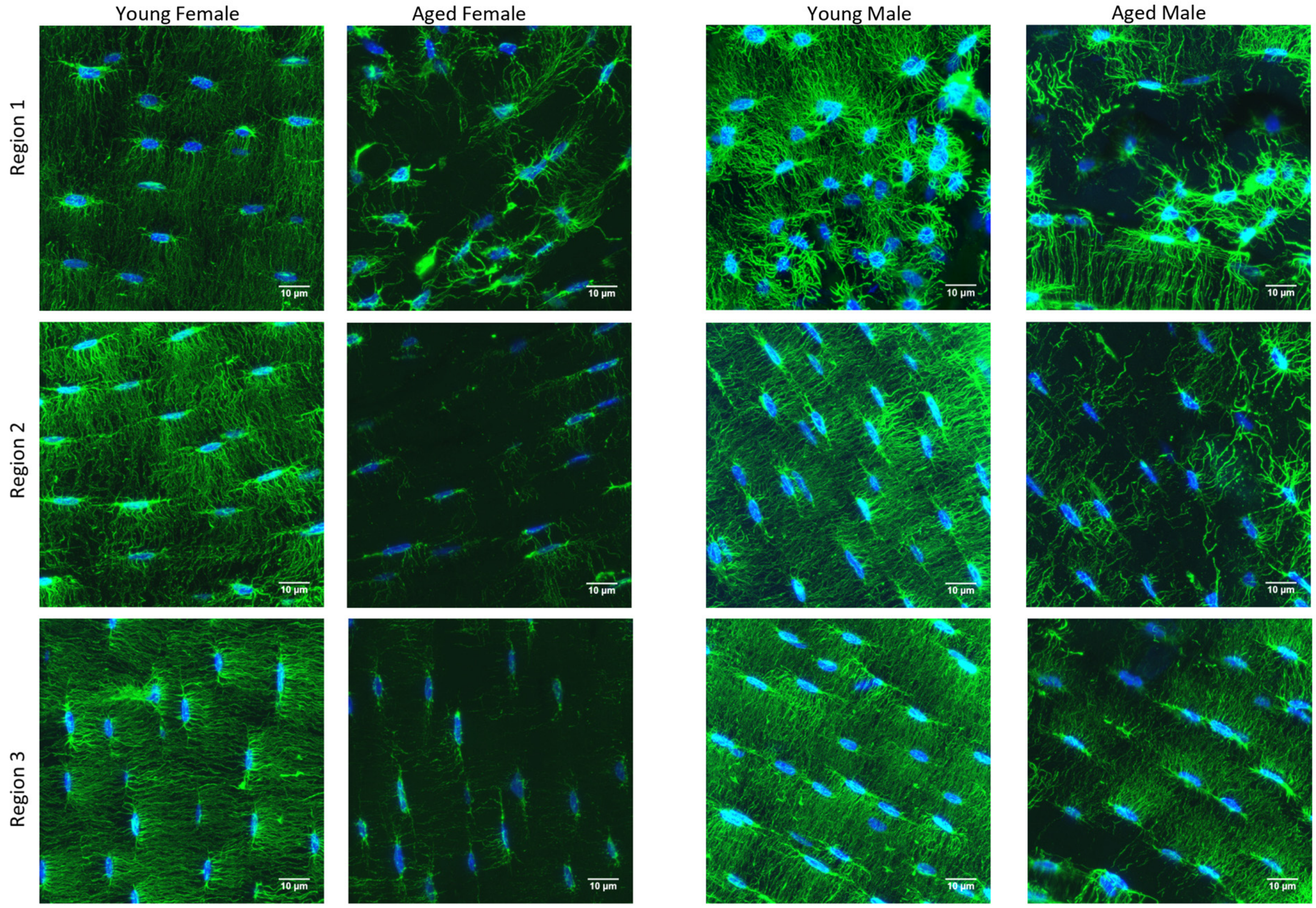

Supplementary Figure S1. Examples of confocal images of phalloidin (green) and DAPI (blue) stained thick sections from the midshaft of the femur in young and aged male and female mice (Z-maximal projections of 250 planes [32.5 µm], imaged at 100x magnification). Images are shown from the three different locations on a young and aged femoral midshaft section to illustrate the variation between the three imaging regions. Note that overall the aged bones show reduced dendrite connectivity compared to the young of each gender. Bar = 10µm.

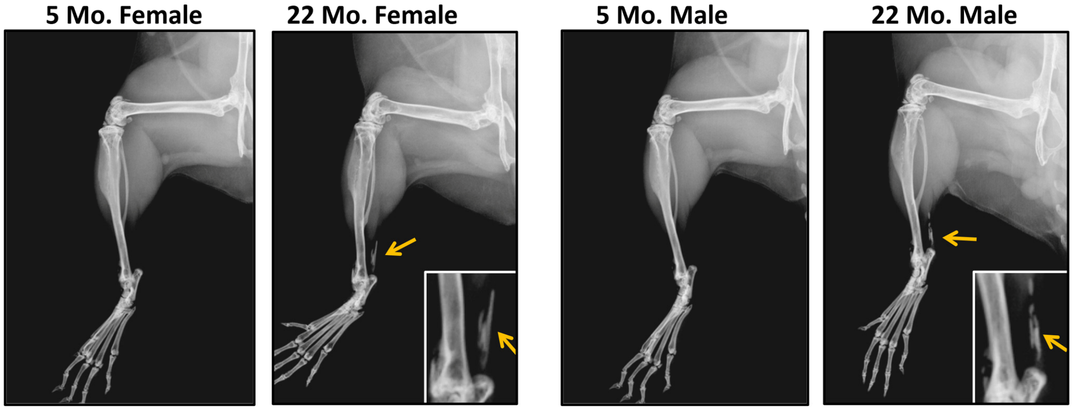

Supplementary Figure S2. X-ray images of mouse hindlimbs showing the calcified tendons in the aged female and male mice (yellow arrows and enlarged insets). This tendon calcification was seen in 100% of the aged mouse specimens.
